# The effect of aerobic exercise on interoception and cognitive function in healthy university students: a non-randomized controlled trial

**DOI:** 10.1186/s13102-021-00332-x

**Published:** 2021-08-28

**Authors:** Yusaku Amaya, Tetsuya Abe, Kenji Kanbara, Hisaharu Shizuma, Yasushi Akiyama, Mikihiko Fukunaga

**Affiliations:** 1 Department of Psychosomatic and General Internal Medicine, Kansai Medical University, 2-5-1 Shinmachi, Hirakata, Osaka 573-1010 Japan; 2 Faculty of Rehabilitation, Shijonawate Gakuen University, Osaka, Japan; 3 Psychosomatic Medicine, Clinical Psychology, Faculty of Medicine, Kagawa University, Kagawa, Japan; 4 Department of Education, Kyoto College of Medical and Health, Kyoto, Japan

**Keywords:** Interoception, Aerobic exercise, Cognitive function, Heartbeat tracking task, Decision-making

## Abstract

**Background:**

Interoception refers to the body’s physiological responses that occur in response to emotions. This phenomenon influences decision-making, an important cognitive ability that affects the maintenance of an exercise routine. However, it is controversial whether interoception is a reliable measure of an individual’s traits or their response to emotion. Given this evidence, we hypothesized that performing an exercise with positive feelings could improve interoception and that the rational decision-making capabilities acquired by improved interoception would, in turn, help in maintaining an exercise routine. Persistent aerobic exercise is essential for improving cognitive and musculoskeletal function in the long term. Therefore, we aimed to investigate changes in interoception during moderate-intensity aerobic exercise at a level that might potentially improve cognitive function.

**Methods:**

We devided 48 healthy university students into an exercise group (n = 37) and a control group (n = 11). The control group did not perform any exercises, while the exercise group performed bench step exercises at an intensity of 50% of heart rate reserve for 30 min a day, three times a week, for three months. We assessed their cognitive function by measuring their auditory information/working memory processing speed using a paced auditory serial addition task (PASAT) and evaluated their interoceptive accuracy (IA) using a heartbeat tracking task at baseline and 1, 2, and 3 months after the start of the exercise intervention.

**Results:**

There was a significant positive correlation between IA and PASAT scores at baseline. However, exercise did not lead to a significant increase in PASAT scores of the exercise group as compared with the control group. IA scores increased at 2 and 3 months after the start of exercise only in the exercise group.

**Conclusions:**

This preliminary study showed an improvement in interoception after persistent moderate-intensity aerobic exercise. We believe that exercise-induced improvement of interoception may facilitate exercise maintenance through improved cognitive function. Statistical analysis did not explain the non-uniformity of sample sizes, therefore, future studies should have larger sample sizes with equal subjects in each group to allow for better comparability and generalizability.

*Trial registration*: UMIN, UMIN000042891. 04/01/2021, retrospectively registered.

## Background

Persistent aerobic exercise is known to be a key intervention for enhancing an individual’s musculoskeletal and cardiorespiratory health, but it also plays an important role in improving cognitive functions, such as memory, information processing speed (IPS), and decision-making [1, 2]. The basal ganglia, which control subtle movement in physical exercise, form a loop network with the prefrontal cortex to influence higher-order brain functions such as learning or cognition [3, 4].

The dropout rate, i.e., the rate of dropping out of an exercise routine, influences the beneficial effect that exercise can have on physical and mental health [5, 6]. The rate depends on an individual’s physical, environmental, social, and psychological parameters. Decision-making is a particularly important cognitive ability that can determine whether an individual maintains or discontinues their exercise routine. According to the somatic marker hypothesis (SMH) developed by Antonio Damassio, experiencing an emotional somatic awareness of a preceding external stimulus influences an individual’s decision-making ability by activating the anterior cingulate cortex (ACC), insular cortex (IC), ventromedial prefrontal cortex, and/or ventrolateral prefrontal cortex in their brain [7]. This means that rational decision-making tendencies should be able to perceive the physiological as well as psychological changes induced by exercise.

Interoception is a phenomenon wherein physiological information, such as heart rate and gastrointestinal motility, is perceived by getting relayed to the ACC and IC via the endocrine, immune, and autonomic nervous systems [8]. Psychological conditions are strongly related to interoception [9, 10] and neurological connections between emotion-related brain activity and interoception can be visualized using electroencephalograms [11] and functional magnetic resonance imaging (MRI) [12]. Healthy individuals with high interoception have reportedly shown a better decision-making performance [13, 14]. These results indicate the similarity between an emotional somatic experience and interoception. Interoception is widely considered a stable index that reflects a fixed individual trait. Abnormal interoception is associated with sustained panic attacks [15] and reduced sympathetic reactivity during periods of mental stress [16]. In psychosomatic medicine, individuals with alexithymia show poor awareness of their emotions, and the psychological characteristic is associated with the development of psychosomatic diseases [17]. Impaired interoception may potentially be associated with alexithymia [18, 19]. In conjunction with this, interoception is also considered as a status index that interacts with psychological conditions. Altered interoceptive states are associated with depression and anxiety [20, 21]. Mental training can improve interoception in healthy individuals [22, 23]. Interoceptive training reduces symptoms in patients with somatoform disorders [24] and facilitates increased rationality of decision-making in healthy individuals [25]. These results show a bidirectional association between interoception and psychological states.

Based on this evidence, we hypothesized that there might be an interaction between maintaining aerobic exercise and improved interoception. Annesi reported an increase in positive feelings and self-motivation during a 12-week exercise for weight management [26]. It is commonly assumed that a positive feeling induced by maintaining exercise improves interoception, and in turn, the increased rationality in decision-making that is acquired by improved interoception facilitates the maintenance of an exercise routine. To clarify this association further, we aimed to identify the changes in interoception induced by exercise at a level that was expected to improve cognitive function in healthy university students.

## Methods

### Participants

We recruited 48 university students from Shijonawate Gakuen University, after excluding those who exercised frequently. All students underwent health checkups, and we further excluded those with any type of medical disorder. Following the study protocol that was approved by the ethics committee of Kansai Medical University Hospital, we obtained written informed consent from all participants. We allocated the participants non-randomly into two groups based on their classes at the university; however, most of students in one class declined to participate in the study, due to their tight schedule. The exercise group included 37 participants and the control 11 participants. Four participants in the exercise group experienced difficulty in continuing with the task due to changes in their circumstances; thus, the exercise group finally included 33 participants .

### Procedure

**Fig. 1:**
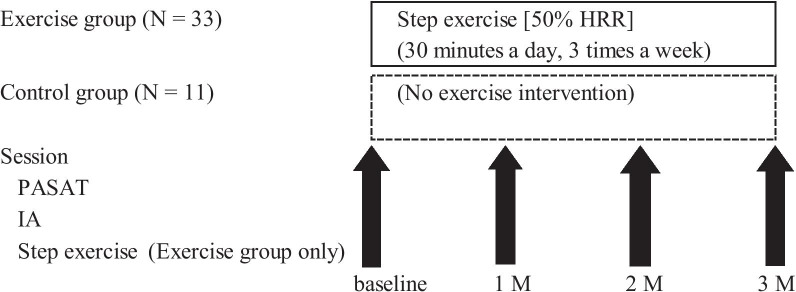
Study protocol. The session consists of the PASAT and IA measurements and the step exercise training in this order. Both the exercise and control groups executed the sessions at baseline and 1, 2, and 3 months after the start of the exercise intervention. N,  number; HRR, heart rate reserve; PASAT,  paced auditory serial addition task; IA,  interoceptive accuracy; M, month

We conducted this research from July 2018 to December 2018. Figure 1 represents the study protocol schematically. All the participants sat in a comfortable chair in a quiet room with a constant temperature of 25 °C. We first measured cognitive function and then interoception. In the exercise group, a physical therapist instructed the participants to perform stepping exercises for 30 min immediately after the measurements; from that day, the participants started the exercise once a day, three times a week, for 3 months at home. The control group was asked to spend time as usual, without any exercise-based intervention. We repeated the measurements and the instruction of exercise once a month for 3 months (a total of four times). We requested both groups not to make an extra habit of exercising in their leisure time during the 3-month intervention. No adverse events occurred in either group.

### Assessment

#### Cognitive function

The paced auditory serial addition task (PASAT) is an assessment tool for IPS [27]. A tester auditorily presented 61 single-digit numbers at one-second intervals, and the participants were required to orally answer with the sum of the two consecutive numbers as soon as possible. The percentages of correct answers was calculated as the score.

#### Interoception

We measured IA using a heartbeat tracking task derived from a mental tracking task [28]. Although its validity remains unclear due to beliefs related to heart-rate counting [29], this test has confirmed high reliability [30]. The participants wore an electrocardiograph device with electrode pads attached to their left chest (myBeat heart rate sensor, WHS-2 Union Tool Co., Ltd., Tokyo, Japan). While the electrocardiograms continued to record, after a 10-minute adaptation period, they counted their heart rates in random order during three different periods (25, 35, and 45 s) by closing their eyes and not taking their pulse. IA scores were calculated using the following formula:

$${\text{IA = 1/3}}\sum {{\text{[1}} - {\text{(|recorded}}\;{\text{count}} - {\text{perceived}}\;{\text{count|)/recorded}}\;{\text{count]}}}$$

The IA values ranged from 0 to 1, with values closer to 1 indicating a higher heartbeat tracking accuracy.

### Exercise intervention

High-intensity exercise improves both cognitive and mental function [31, 32], whereas moderate-intensity exercise is known to improve cognitive and mental function [33, 34] through neurogenesis and neuroendocrine responses [35, 36]. We adopted the 50% heart rate reserve (HRR) task, which is considered to be a moderate-intensity exercise as per ACSM’s Guidelines for Exercise Testing and Prescription [37], and employed a previously-reported step exercise using a low-height bench [33]. For the former intensity, we calculated the target heart rate using the formula:

$${\text{[(maximum}}\;{\text{heart}}\;{\text{rate}} - {\text{resting}}\;{\text{heart}}\;{\text{rate)}} \times {\text{target}}\;{\text{exercise}}\;{\text{intensity]}} + {\text{resting}}\;{\text{heart}}\;{\text{rate}}, {\text{where}}\;{\text{the}}\;{\text{maximum}}\;{\text{heart}}\;{\text{rate}} = \;{\text{220}} - \;{\text{age}}$$

The latter step exercise has two advantages: first, the low height of the step (20 cm) makes it a safer and easier exercise, and second, the step can be easily substituted by stairs, which makes it possible to perform the exercise indoors without being influenced by an external environment. During the step exercise, the examiner, a well-trained physical therapist, monitored the participants’ heart rates using a wearable electrocardiograph device and trained them to exercise at a consistent level of intensity to maintain a 50% HRR value for 30 min. The examiner counted the steps per minute during that time and informed the participants about the number of steps they had achieved to serve as a benchmark for them to perform the exercise with the same intensity at home.

### Statistical analysis

Values are presented as mean ± SD. The Shapiro-Wilk test was used to test normality. We used a Chi-square test with Yates’ correction to compare the sex distribution, and the *t*-test to age, BMI, resting heart rate, maximal heart rate, and the calculated 50% HRR between the exercise and control groups. Levene’s test was performed to assess the homoscedasticity of the *t*-test and Welch’s *t*-test was applied for unequal variances. The correlation between IA and PASAT scores in the baseline phase in all participants was examined using Spearman’s rank correlation coefficient. For the comparison of IA and PASAT scores between the exercise and control groups, we conducted a two-way repeated-measures analysis of variance (ANOVA) with “points” (four levels: baseline and 1, 2, and 3 months after the start of exercise intervention) as the within-subject factors and “groups” (two levels: the exercise and control groups) as the between-subject factors. We also performed Mauchly’s sphericity test to assess the homoscedasticity of the two-way repeated-measures ANOVA and applied the Huynh-Feldt correction when the sphericity assumption is violated. Subsequently, when the ANOVA results were significantly different, we used a Bonferroni’s correction as a multiple comparison test. A post hoc power analysis was performed using G*Power 3.1 [38]. All statistical analyses were performed using SPSS Statistics for Windows (version 20.0; SPSS Inc., Chicago, IL, USA). The α level was set at 0.05.

## Results

On comparing the exercise and control groups, we found no significant differences based on sex, age, BMI, resting heart rate, maximal heart rate, and the calculated 50% HRR (Table 1).

**Table 1:** Demographic data of participants

	Exercise (N = 33)	Control (N = 11)	*P* value
Female (%)	9 (27.3%)	5 (45.5%)	0.29
Age (mean ± SD, years)	21.1 ± 1.1	21.4 ± 0.5	0.45
BMI (mean ± SD, kg/m^2^)	22.4 ± 3.2	22.3 ± 3.6	0.95
HR-max (mean ± SD, bpm)	198.89 ± 0.98	198.64 ± 0.48	0.42
HR-rest (mean ± SD, bpm)	69.51 ± 9.36	73.27 ± 8.97	0.25
50% HRR (mean ± SD, bpm)	134.07 ± 5.53	137.23 ± 8.32	0.15

N, number; SD, ,standard deviation; BMI, Body mass index; HR, heart rate; HR-rest, ,resting heart rate; HR-max, maximal heart rate; HRR,  heart rate reserve

### Correlation between the IA and PASAT scores

**Fig. 2:**
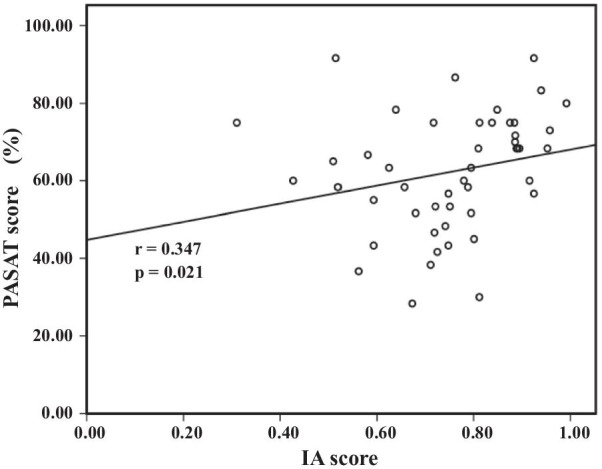
Correlation between IA and PASAT in all participants at baseline. IA, interoceptive accuracy; PASAT,  paced auditory serial addition task

The baseline IA and PASAT scores in all participants were 0.76 ± 0.15 and 65.07 ± 14.92, respectively (n = 44). Figure 2 shows a scatter diagram of the IA and PASAT scores in all participants. Before the intervention, the IA and PASAT scores showed a significant positive correlation (r = 0.347, *p* = 0.021). A post hoc power analysis revealed an effect size of 0.35 and a power of 0.68.

### Information processing speed

**Fig. 3:**
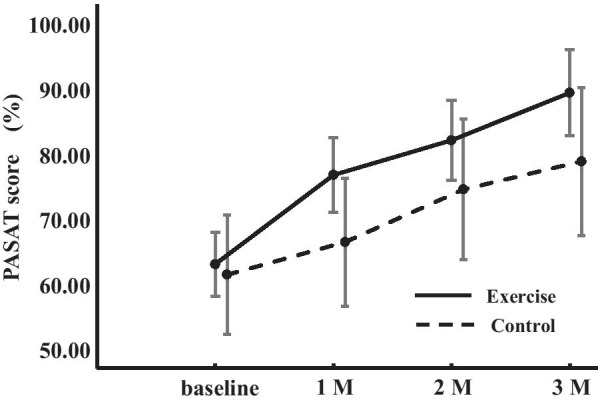
PASAT change induced by exercise in the exercise and control groups. PASAT, paced auditory serial addition task; M,  month. Error bars represent 95% confidence intervals

Figure 3 represents the change in PASAT scores across the four points of the study (at baseline and at 1, 2, and 3 months after the start of the exercise intervention ) in both subject groups. The two-way ANOVA indicated that the point main effects were significant [F(3,126) = 25.853, *p* < 0.001, η^2^ = 0.381]. A post hoc power analysis revealed an effect size of 0.87 and a power of 1.00. Neither the point-group interaction [F(3,126) = 0.792, *p* = 0.500, η^2^ = 0.019] nor the group main effects [F(1,42) = 2.193, *p* = 0.146, η^2^ = 0.050] were significant. In the exercise group, the PASAT scores were significantly higher at 3 months after the start of exercise than at baseline (*p* < 0.001), at 1 month after the start of exercise (*p* < 0.001), and at 2 months after the start of exercise (*p* = 0.001). The scores at the 2-month mark were significantly higher than those at baseline (*p* < 0.001) and 1 month (*p* = 0.011). Finally, the 1-month scores were significantly higher than the baseline (*p* < 0.001). Meanwhile, in the control group, the PASAT scores were higher at the 3-month and 2-month marks than at baseline (*p* = 0.001 and 0.011, respectively) or 1 month after the start of exercise (*p* = 0.004 and 0.015, respectively).

### Interoception

**Fig. 4:**
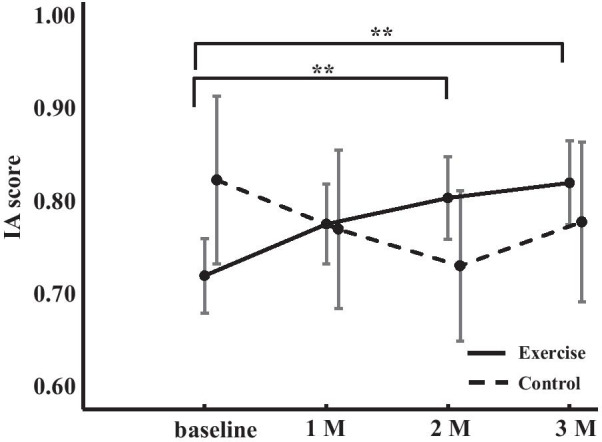
IA change induced by exercise in the exercise and control groups. IA, interoceptive accuracy; M,  month. Error bars represent 95% confidence intervals. **p < 0.01 (compared in the exercise group)

We compared the changes in IA scores over time between the exercise and control groups using a two-way ANOVA (Fig. 4). There were no significant main effects of either the point [F(3,126) = 0.716, *p* = 0.515, η^2^ = 0.017] or the group [F(1,42) = 0.015, *p* = 0.903, η^2^ < 0.001], however, the point-group interaction was significant [F(3,126) = 3.334, *p* = 0.032, η^2^ = 0.074]. A post hoc power analysis revealed an effect size of 0.28 and a power of 1.00. This simple main effects test revealed that IA scores were significantly different only in the exercise group between the baseline and 2-month period (*p* = 0.014) and between the baseline and 3-month period after the start of exercise (*p* = 0.003).

## Discussion

In this preliminary study, we investigated the changes in IA to understand the mechanisms that facilitate the maintenance of an exercise routine in healthy individuals. There was a significant positive correlation between IA and cognitive function before the exercise intervention. The exercise and control groups exhibited different patterns of changes in IA, and IA increased significantly after 2–3 months of exercise.

Our study demonstrated the relation between exercise and interoception. Williamson et al. reported a significant increase in blood flow volume in the ACC and IC during a hand exercise task using MRI and single-photon emission computed tomography [39], and this indicates the existence of a neurological network in the regions that govern movement and interoception in the central nervous system. Our study is the first report to reveal these neuroimaging findings by clinically quantifying interoception, which is a psychological index. Using functional MRI, Critchley et al. showed that, when attention is paid to a heartbeat tracking task, the brain is activated not only in the ACC and IC but also in the primary motor cortex and supplementary motor area, which govern voluntary movements [40]. This result suggests that focusing on interoception activates motor areas of the brain as well as the pathways of interoception. Experiments that involved adults pedaling a cycle ergometer [41] and children running for 6 min [42] revealed that interoception influences the self-regulation of physical activity. These studies underpin our hypothesis that the interaction between interoception and maintaining exercise reduces the dropout rate from an exercise routine.

In this study, we chose persistent moderate-intensity exercise as a physical intervention. Initiating and maintaining exercise therapy is challenging. Individuals who drop out often show lesser enjoyment of exercise at baseline [43], and motivation to exercise relates to exercise enjoyment and positive affect [44]. These reports reinforce our hypothesis that exercise enjoyment increase the rational decision-making for maintaining exercise. Exercise enjoyment increases during moderate-intensity exercise, but not high-intensity exercise [43]. Higher-intensity exercise tends to induce non-utilitarian decision-making [45]. Acute exercise increases cognitive function, and which is not induced by decision-making capability [46]. These indicate the importance of both the intensity and the duration of exercise intervention for the improvement of interoception.

Our results outlined neurological networks showing evidence that exercise improves cognitive function. We also investigated another network associated with the link between emotions and decision-making capabilities, known as the SMH. Our result shows that interoception increased only after 2–3 months of moderate-intensity exercise and congitive function might have increased after 1 month of the exercise. We consider that exercise increases cognitive function by the activation of two different brain networks and persistent moderate-intensity exercise stimulates both networks to improve cognitive function. Interoception may be used as an index to reflect the activation of the latter network. As we elucidate that interoception does not sufficiently increase within a short period, it is necessary to assess the effect of high-intensity exercise on interoception.

## Limitations

First, there was a large difference in the sample size of our subject groups. We allocated our subjects non-randomly based on their classes at the university, and because of a challenging curriculum in one class, most of the students declined to participate in the study, which resulted in non-uniform group sizes. We ensured that the assumption of normality of indexes was not violated in each group and that there was homoscedasticity between IA and PASAT in both groups. The G*Power software also detected sufficient statistical power. However, this software cannot take the unequal group sizes into account, and these statistical results do not address this issue. In future studies, it is necessary to increase the number of participants in general and also achieve uniformity in group sizes.

Second, we could not identify exercise-induced improvements in cognitive function or the causal relationship between the changes in interoception and cognitive function. The repetitive use of PASAT impeded our assessment of cognitive function, which was the main limitation. While PASAT is an established tool for measuring IPS, its practice effects have also been reported [47]. In our data, PASAT increased slowly and steadily in both subject groups, which can be caused by the practice effect. The other limitation was related to the subjects’ characteristics. PASAT was originally developed for patients with neurological dysfunction. Our participants were healthy, young, and intelligent individuals, and each of these factors can independently increase the IPS [48, 49] and may potentially lead to a ceiling effect. Therefore, these effects might obscure the results linked to PASAT-changes induced by exercise intervention. However, PASAT has been employed in healthy young subjects [50, 51], and our results also showed a cross-sectional correlation between PASAT and IA before the intervention, which is in line with previous research [40]. The PASAT reference value for healthy Japanese individuals in their twenties is 57.7 ± 14.71 [52], which is similar to what we reported. Our results support the validity of using PASAT to evaluate our participants. In healthy young subjects, moderate-intensity exercise is proven to improve cognitive function [35, 36], and our protocol is expected to increase PASAT.

Third, there is no objective data on the participants’ activities during their daily lives or while performing the exercises. However, we chose participants whose base activities were expected to be low to elucidate the effects of the intervention most effectively.

Finally, although the present study included participants of both sexes, several studies have reported sex differences in interoception [53]. Future research should verify our findings across both sexes individually with a larger sample size from the general population.

## Conclusions

This preliminary study showed the improvement of interoception by persistent aerobic exercise at a level that might potentially increase cognitive function. Although we failed to effectively demonstrate the link between the changes induced by exercise in both interoception and cognitive function, our findings supported the hypothesis that exercise-induced improvement of interoception facilitated improved maintenance of an exercise routine as a result of improved cognitive function. Although our statistical analyses were appropriate, both the subject groups comprised different numbers of participants. In future studies, it is necessary to test a larger sample size and to ensure that the subject group sizes are uniform.

## Data Availability

Data of participants who agreed to the public distribution of data are available from the corresponding author upon reasonable request.

## Abbreviations

ACC: Anterior cingulate cortex
BMI: Body mass index
HRR: Heart rate reserve
IA: Interoceptive accuracy
IC: Insular cortex
IPS: Information processing speed
MRI: Magnetic resonance imaging
PASAT: Paced auditory serial addition task
SMH: Somatic marker hypothesis
